# Supplementary material for: Metformin intervention ameliorates AS in ApoE-/- mice through restoring gut dysbiosis and anti-inflammation
Source: PLoS One. 2021 Jul 15;16(7):e0254321. doi: 10.1371/journal.pone.0254321 (PMC8282009; doi:10.1371/journal.pone.0254321)
Supplement: Attachment — Submitted filename: Response to Reviewers.docx [file pone.0254321.s002.docx]

Dear editor,

Thank you for your kind letter. Thanks for your constructive suggestions and the reviewers’ valuable comments on our manuscript. We have revised the manuscript in accordance with the comments, and carefully proofread the manuscript to minimize typographical, grammatical, and bibliographical errors.

We have uploaded 3 files (Response to Reviewers, Revised Manuscript with Track Changes, Manuscript) to the submission system. All alterations in the manuscript were marked with red color.

**Response to editor comments**

Thank you for your constructive suggestions. According to all of your kind comments, our manuscript has been carefully revised. Please check them.

a) Journal Requirements: Please review your reference list to ensure that it is complete and correct. If you have cited papers that have been retracted, please include the rationale for doing so in the manuscript text, or remove these references and replace them with relevant current references. Any changes to the reference list should be mentioned in the rebuttal letter that accompanies your revised manuscript. If you need to cite a retracted article, indicate the article’s retracted status in the References list and also include a citation and full reference for the retraction notice.

**Reply:** We really appreciate your careful advice. We have carefully checked the Reference list to make sure that there’s no retracted paper in the Reference. Furthermore, we also downloaded the Plos endnote style to unify the reference format and checked the reference format manually to make sure our references style meet PLOS ONE’s publication standards. All alterations in Reference section of the manuscript were marked with red color.

b) While revising your submission, please upload your figure files to the Preflight Analysis and Conversion Engine (PACE) digital diagnostic tool, https://pacev2.apexcovantage.com/. PACE helps ensure that figures meet PLOS requirements. To use PACE, you must first register as a user. Registration is free. Then, log in and navigate to the UPLOAD tab, where you will find detailed instructions on how to use the tool. If you encounter any issues or have any questions when using PACE, please email PLOS at figures@plos.org. Please note that Supporting Information files do not need this step.

**Reply:** Thank you for your comments. According to your suggestion, we have uploaded all the figures to the PACE tools, and make sure all the figures meet PLOS requirements.

**Response to Reviewer #1**

1) The ZO-1 quantification from immunofluorescence figures of Fig 7A looks not very consistent with the barplot of Fig 7B.

**Reply:** We really appreciate your careful comments. Zonula occludens-1 (ZO-1) protein, an epithelial tight junction protein, is a biomarker of intestinal permeability. With the accelerating progression of atherosclerosis, the expression of ZO-1 showed a notable decrease. In our study, in Fig 7A and 7B, compared with the CON group, the ZO-1 expression in the AS group was significantly decreased, and it could be restored by MET intervention. Thank you for your valuable advice, according to your suggestion, we have carefully checked the data in Figure 7 and found that there’s no wrong in ZO-1^+^ area/intestinal area in the Fig 7. The dataset for Fig 7B has been provided in the following table. Thank you for your rigorous attitudes.

| ZO-1+ area / intestinal area | CON | AS | AS+MET |
| --- | --- | --- | --- |
|  | 0.153 | 0.103 | 0.149 |
|  | 0.149 | 0.114 | 0.158 |
|  | 0.186 | 0.097 | 0.133 |
| Mean | 0.1627 | 0.1047 | 0.1467 |
| Std. Deviation | 0.02031 | 0.008622 | 0.01266 |

2) The subtitle of "MET attenuated the integrity of gut mucosa" should be revised as "MET restored the integrity of gut mucosa" to avoid misunderstanding.

**Reply**: We really appreciate for your kind comments. In our manuscript, we have replaced the subtitle of "MET attenuated the integrity of gut mucosa" as "MET restored the integrity of gut mucosa", in accordance with your constructive suggestion. The changes in the manuscript were marked with red color.
